# Effect of Transcranial Alternating Current Stimulation on Prevention of Postoperative Pain After Video‐Assisted Thoracic Surgery: A Randomized Controlled Trial

**DOI:** 10.1155/prm/6121920

**Published:** 2026-07-21

**Authors:** Jin-Jin Yang, Sai Chen, Zhi-Hao Li, Ni Du, Lei Lei, Jian-Jun Yang

**Affiliations:** ^1^ Department of Anesthesiology, Pain, and Perioperative Medicine, The First Affiliated Hospital of Zhengzhou University, No. 1 East Jianshe Road, Zhengzhou 450002, China, zzu.edu.cn; ^2^ Henan Province International Joint Laboratory of Pain, Cognition and Emotion, Zhengzhou, China

**Keywords:** postoperative pain, transcranial alternating current stimulation, tACS, video-assisted thoracoscopic surgery

## Abstract

**Introduction:**

Transcranial alternating current stimulation (tACS), which can noninvasively entrain oscillatory brain activity, has attracted scientific attention as a possible technique to control pain. However, there is a scarcity of studies investigating the preventive effect of tACS on postoperative pain.

**Methods:**

This double‐blind, randomized, sham‐controlled trial enrolled 72 patients undergoing elective video‐assisted thoracoscopic surgery (VATS). Patients were randomly allocated (1:1) to receive a single 20‐min session of α‐tACS on the primary somatosensory cortex (S1) or sham stimulation postoperatively. The primary outcomes were postoperative numerical rating scale pain scores and opioid consumption at 24 h postoperatively. Secondary outcomes included cumulative opioid consumption within 48 h and Quality of Recovery‐15 (QoR‐15) score. Adverse events were also assessed.

**Results:**

The tACS group exhibited significantly lower resting pain scores versus the sham group (*β* = −0.49, 95% confidence interval [CI], −0.78 to −0.20, *p* = 0.001) and lower movement pain scores versus the sham group (*β* = −0.45, 95% CI, −0.84 to −0.06, *p* = 0.025), though cumulative opioid consumption showed no difference at 24 h (median difference [MD] = 1.0 mg; 95% CI, −2.3 to 2.5; *p* = 0.76) and 48 h (MD = 3.3 mg; 95% CI, −0.6 to 9.1; *p* = 0.10) postoperatively. Additionally, the area under the curve for resting pain over 2–24 h (AUC_2–24 h_) and 2–48 h (AUC_2–48 h_), as well as the AUC_2–48 h_ of movement pain scores, were significantly lower in the tACS group. Moreover, QoR‐15 scores and adverse events were also comparable.

**Conclusion:**

For patients undergoing VATS, a single α‐tACS treatment targeting the bilateral S1 regions yielded a statistically significant yet modest reduction in postoperative pain. However, no significant decrease in postoperative opioid consumption was observed, and further research is warranted.

**Trial Registration:** Chinese Registry of Clinical Trials: ChiCTR2300078723

## 1. Introduction

Pain is an unpleasant sensory and emotional experience [1], surgery trauma often causes severe postoperative pain in patients, and studies have reported that even with video‐assisted thoracoscopic surgery (VATS), 38% of patients experience clinically relevant moderate‐to‐severe pain within the first 24 h postoperatively [2]. Inadequate postoperative pain control is associated with negative emotions, increased postoperative complications, prolonged hospital stays [3–5], and even progression to chronic pain within 10–50 months [6], all of which severely impair patients’ postoperative quality of life. Opioids are commonly used for acute postoperative pain relief but carry inherent risks of side effects, including respiratory depression, nausea, vomiting, and constipation [7–10]. While paravertebral nerve block is widely used as part of multimodal analgesia, it is invasive and sometimes complicated by bleeding, infection, and nerve injury [11]. Thus, it is necessary to explore new noninvasive interventions that can relieve postoperative pain and reduce opioid dependence.

Over the past two decades, neuromodulation techniques have gradually become a new direction of pain treatment [12]. Transcranial alternating current stimulation (tACS), an emerging noninvasive brain stimulation technique, modulates task‐related neural rhythms through frequency‐specific exogenous electrical fields, as demonstrated by neuronal models [13], animal experiments [14, 15], and human studies [16]. This process, termed neural entrainment (phase synchronization of endogenous neural oscillations in targeted brain regions with the stimulation signal), is the core mechanism underlying tACS’s neuromodulatory effects. Recent studies have demonstrated the promising efficacy of tACS in relieving experimental pain [17] and chronic pain such as migraine, fibromyalgia, and neuralgia [18, 19], highlighting its potential as an innovative therapeutic alternative for pain disorders. Nevertheless, to date, the efficacy of tACS for alleviating postoperative acute pain and reducing opioid consumption remains unclear, and the relevant studies are lacking. The primary somatosensory cortex (S1) is a key brain region involved in pain perception and modulation, responsible for the topological representation of somatic sensations and direct participation in the spatial localization and intensity coding of nociceptive inputs [20], and preliminary studies [17, 21] suggest that alpha‐frequency tACS over the S1 exerts analgesic effects.

Thus, the current study aimed to evaluate the efficacy of alpha tACS over S1 as an adjunctive analgesia for the prevention of postoperative pain in patients undergoing VATS.

## 2. Material and Methods

### 2.1. Study Design and Ethics

The study was a double‐blinded, randomized, sham‐controlled, perprotocol clinical trial conducted at First Affiliated Hospital of Zhengzhou University in China. The trial was approved by the Ethics Review Committee (2023‐KY‐1142‐003) of the First Affiliated Hospital of Zhengzhou University and prospectively registered. The study followed the Declaration of Helsinki principles, and written informed consent was obtained from all participants.

### 2.2. Participants

Patients who were scheduled to receive VATS for lung cancer resection in the First Affiliated Hospital of Zhengzhou University after December 2023 were eligible for screening. Taking into account the differences in patient management between groups of surgeons, we only recruited patients from the same surgical group.

Eligibility criteria were as follows: (1) 18 ≤ age ≤ 80 years old; (2) American Society of Anesthesiologists physical status Class I–III; and (3) patients undergoing elective VATS for lung cancer resection. Exclusion criteria were as follows: (1) contraindications to transcranial electrical stimulation (implantation of electronic devices in the body, such as pacemakers or other metal devices, cavities or fissures in the skull, or local skin damage or inflammation); (2) current pregnancy or lactation; (3) history of neurosurgery, epilepsy, or psychiatric disorders; (4) patients with a history of chronic pain, long‐term use of analgesia or sedative drugs, or alcohol abuse; (5) patients with impaired liver and kidney function (ALT > 80 U/L and/or AST > 80 U/L, total bilirubin > 34.2 μmol/L, or preoperative creatinine level > 133 μmol/L); (6) BMI ≥ 40 kg/m^2^; (7) history of long‐term use of anticoagulant drugs, or coagulation abnormalities; (8) patients who are allergic to the drugs required in the study or refuse to use the intravenous patients‐controlled analgesic pump; and (9) patients who refuse to sign the informed consent form.

Patients were screened on the day before surgery. The baseline characteristics of the participants were recorded. Additionally, preoperative sleep quality, anxiety, and depressive symptoms were comprehensively evaluated using the Pittsburgh Sleep Quality Index and Hospital Anxiety and Depression Scale, respectively. Moreover, patients were educated on the proper usage of the patient‐controlled intravenous analgesia pump.

### 2.3. Randomization and Blinding

A simple randomization list was generated using a computer‐generated 1:1 assignment by one researcher who was not involved in the trial procedures, follow‐up, and analysis. For assignment concealment, the assignment information was placed in a sealed opaque envelope marked with the coding order, kept by a dedicated researcher. The study implementer coded the study subjects in order of enrollment. The investigator opened the envelope in the order of coding and administered transcranial electrical stimulation or sham stimulation depending on the group, and the researcher was not involved in the other parts of the study. None of the patients, anesthesiologists, or follow‐up staff were aware of the group assignments.

### 2.4. Procedures

#### 2.4.1. General Anesthesia Management

All patients were strictly fasted for at least 6 h and deprived of water for 2 h. A single‐point paravertebral nerve block on the operative side was administered in the preoperative preparation room. All blocks were performed by the same experienced anesthesiologist using an ultrasound‐guided in‐plane technique at the T4 vertebral level, with 20 mL of 0.25% ropivacaine injected per patient.

Intraoperative management was standardized to a general anesthetic. Prophylactic penehyclidine (0.01 mg/kg) was given to inhibit gland secretion. Anesthesia was induced with etomidate (0.2–0.3 mg/kg), alfentanil (25–75 μg/kg), and cisatracurium (0.6–0.8 mg/kg), and endotracheal intubation was performed. To reduce the dosage and side effects of a single drug and leverage the synergistic effect of two types of anesthetics, anesthesia maintenance was performed using a combination of intravenous and inhalation balanced anesthesia, including sevoflurane (1%–2%), propofol (2–4 mg/kg/h), remifentanil (0.1–0.3 μg/kg/min), and intermittent bolus of cis‐atracurium (0.15 mg/kg). End‐tidal carbon dioxide was maintained between 35 and 45 mmHg, and BIS values were maintained between 40 and 60 intraoperatively. Blood pressure fluctuations were maintained within ±20% of basal blood pressure, and the anesthesiologists selected vasoactive drugs according to their preference. The dual therapy was used to prevent postoperative nausea and vomiting, with dexamethasone 5 mg immediately after anesthesia induction and palonosetron 0.25 mg 20 min before the end of surgery. At the end of the surgery, all medications were discontinued, the patients were transferred to the post‐anesthesia care unit, and the endotracheal tube was removed when the extubation indication was met.

#### 2.4.2. Interventions

All patients received the assigned stimulation immediately after extubation.

The distance between the anatomical landmarks nasion and inion as well as the distance between the preauricular points were measured for each participant, and a cross mark was placed halfway on both lines at the central midline (CZ localization). Then, the EEG cap was placed on the head of the participants. Place two electrodes at the CP3 and CP4 EEG cap positions corresponding to the primary somatosensory cortex (S1) scalp region. Stimulation mode setting: alpha stimulation frequency 10 Hz, stimulation intensity 2 mA, stimulation time 20 min, including 30 s of ascending phase and 30 s of descending phase (Boruikang Technology [Changzhou] Co., Ltd.; machine specification model: NeuStim wireless transcranial electrical stimulation system, which consists of a stimulation host, a splitter, an intelligent synchronization center, electrodes, a cap, a headband, stimulation software, and other components; electrode type: Ag/AgCl electrode; electrode size: 12 (OD) × 6 (ID) mm). The sham stimulation group had stimulation electrodes placed at the same locations. However, electrical current was only administered during a 30‐s ramp‐up and ramp‐down period at the beginning and end of the stimulation to mimic the initial sensation of real stimulation, with no current delivered during the intermediate phase. The impedance levels were kept below 5 k ohms to ensure good contact of the electrodes with the scalp and to ensure that stimulation had not failed. The impedance levels were checked by monitoring them when displayed on the stimulator screen. The researchers who implemented the intervention closely monitored the participants’ vital signs and recorded adverse reactions.

#### 2.4.3. Pain Management and Postoperative Follow‐Up

For the prevention of remifentanil‐induced hyperalgesia and early postoperative acute pain, 10 mg dezocine and 2 g propacetamol hydrochloride were given 20 min before the expected end of surgery. Pain intensity was assessed using a 0–10 point numerical rating scale (NRS). Participants were instructed with standardized instructions that a score of 0 means *no pain* and a score of 10 means *the worst pain imaginable*. If the NRS in the PACU was ≥ 4, oxycodone (0.05 mg/kg) was given for rescue analgesia. Other standardized postoperative analgesia protocols include propacetamol hydrochloride (2 g) every 12 h in the surgical ward and a patient‐controlled intravenous analgesia pump. The parameters of the analgesic pump were set as follows: hydromorphone (0.2 mg/kg) and palonosetron (0.25 mg) were diluted to 100 mL with 0.9% sodium chloride solution; the background dose was set to 1 mL/h, the bolus dose was 2 mL, and the lockout interval was 10 min. If the postoperative NRS pain score was ≥ 4 and the analgesic pump was ineffective, a single intravenous dose of dezocine 5 mg was given for rescue analgesia. Postoperative pain management was implemented by a professional member of the acute pain management team. Follow‐up was conducted at 2 h, 6 h, 12 h, 24 h, and 48 h postoperatively, and the rest and movement NRS pain scores were determined.

### 2.5. Outcomes

The primary outcome was 11‐point NRS pain scores (NRS, 0 = *no pain*, 0 < NRS < 4 [*mild pain*], 4 ≤ NRS < 7 [*moderate pain*], NRS ≥ 7 [*severe pain*], and 10 = *worst pain imaginable*) and cumulative opioid consumption at 24 h postoperatively.

Second outcomes include (1) cumulative opioid consumption within 48 h after surgery; (2) quality of recovery at 24 h and 72 h postoperatively assessed by the 15‐item Quality of Recovery Scale (QoR‐15 scale), which is a patient‐reported outcome measure evaluating recovery after surgery and anesthesia [22]; and (3) occurrence of analgesic‐related side effects and complications related to electrical stimulation.

### 2.6. Sample Size

Postoperative pain score and morphine consumption were predefined as co‐primary endpoints. Given the co‐primary endpoints, sample size calculations were performed for each endpoint individually, and the larger sample size was selected as the reference sample size. The significance level (*α*) was adjusted to 0.025 to control for inflation of Type I error. Postoperative pain scores constituted repeated‐measures data, as each patient was assessed at multiple time points; thus, sample size calculation was conducted using methods for repeated‐measures designs. Based on a preliminary pilot study with a total of 24 patients (12 patients per group), key parameters were set as follows: effect size = 0.3, significance level *α* = 0.025, Type II error probability *β* = 0.2 (corresponding to a statistical power of 1 − *β* = 80%), number of groups = 2, number of measurements = 5, and correlation among repeated measures = 0.5. Calculations indicated that a minimum of 66 patients would be required to detect between‐factors differences in this outcome. According to our postoperative pain management database, the cumulative opioid consumption at 24 h after VATS was 25 ± 6 mg. A reduction in opioid consumption of more than 30% [23] was considered clinically. With the significance level set at *α* = 0.025 and Type II error probability at *β* = 0.2, calculations determined that a minimum of 28 patients would be needed to detect between‐group differences in this parameter. From the two sample size calculations, the larger value (66 patients) was adopted as the reference. Considering an estimated 8% dropout rate, the final planned enrollment for this study was determined to be 72 patients.

### 2.7. Statistical Analysis

SPSS 24.0 and R software were used for statistical analysis. Normally distributed data were represented by mean ± standard deviation, and non‐normally distributed data were represented by median (interquartile range). The Shapiro–Wilk and Levene’s tests were used to evaluate the normality and the homogeneity of variance, respectively. The continuous variables were compared using the *t*‐test. The Mann–Whitney *U* test was used for nonparametric continuous data. Count data were represented by numbers (percentages), and between‐group differences were compared using a chi‐square test or Fisher’s exact test. A two‐sided *p* value < 0.05 was considered statistically significant.

For the primary outcome, given the co‐primary endpoints, controlling the family‐wise error rate at a prespecified significance level (*α*) was essential to avoid inflated Type I error risks. Therefore, the Bonferroni correction was applied to adjust the significance threshold for primary outcome analyses: The original *α* = 0.05 was adjusted to *α* ^′^ = 0.025, which was calculated as *α* divided by the number of primary comparisons (*k* = 2). Postoperative pain scores collected at multiple time points were analyzed via generalized estimating equations (GEEs) to comprehensively evaluate intervention efficacy. A prespecified analytical strategy was adopted for addressing the group‐by‐time interaction effect. If the group‐by‐time interaction effect was not statistically significant, the analysis would focus exclusively on the main effects of group. If the group‐by‐time interaction effect reached statistical significance, post hoc multiple comparisons of the repeated measurement data would be conducted using the Bonferroni correction method. To comprehensively evaluate patients’ cumulative pain burden and sustained analgesic effect, a post hoc analysis was conducted to calculate the area under the curve (AUC) of pain scores over two postoperative time intervals (2–24 h and 2–48 h) using the trapezoidal rule in GraphPad Prism 9 software.

## 3. Results

**FIGURE 1:**
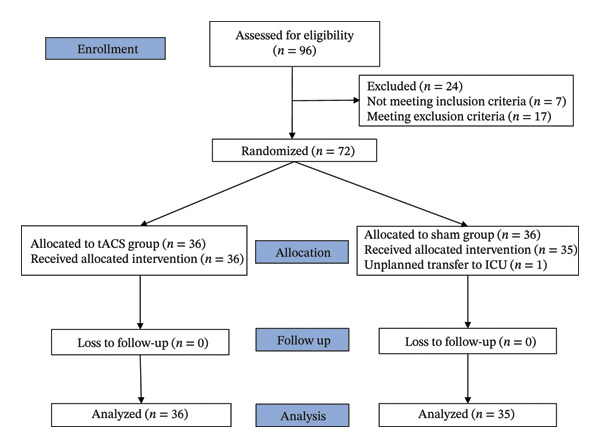
Consort 2010 flow diagram. Abbreviation: tACS, transcranial alternating current stimulation.

From December 2023 to March 2024, a total of 96 patients were assessed for eligibility, of whom 24 patients were excluded before randomization. Overall, 72 patients were randomly allocated, 36 patients per group. In this per‐protocol study, one participant was excluded from the analysis by an unplanned transfer to the ICU, and 71 patients were included in the final analysis (Figure 1).

Overall demographic and intra‐ and postoperative characteristics of patients (Table 1) were comparable between the two groups.

**TABLE 1:** Demographic and intra‐ and postoperative characteristics.

	tACS group	Sham group	*p*
	*N* = 36	*N* = 35	
Age (yr)	55.4 ± 12.1	59.2 ± 12.1	0.20
Male, *n* (%)	15 (41.7)	17 (48.6)	0.56
BMI (kg·m^−2^)	24.6 ± 3.2	23.4 ± 1.8	0.06
ASA classification, *n* (%)			0.91
I	16 (44.4)	15 (42.9)	
II	16 (44.4)	17 (48.6)	
III	4 (11.1)	3 (8.6)	
Hypertension, *n* (%)	11 (30.6)	7 (20.0)	0.31
Diabetes, *n* (%)	5 (13.9)	3 (8.6)	0.71
Coronary artery disease, *n* (%)	2 (5.6)	5 (14.3)	0.26
Cerebrovascular disease, *n* (%)	2 (5.6)	3 (8.6)	0.67
Smoker, *n* (%)	9 (25.0)	6 (17.1)	0.42
Drinker, *n* (%)	10 (27.8)	6 (17.1)	0.28
PSQI	4.5 (2–8)	5 (3–9)	0.31
HADS‐A	4.5 (2–6)	4 (2–5)	0.26
HADS‐D	2 (2–4)	2 (3–4)	0.76
Duration of surgery (min)	133 ± 63	123 ± 50	0.44
Duration of anesthesia (min)	170 ± 65	161 ± 49	0.49
Intraoperative infusion volume (mL)	1200 (825–1400)	1200 (1000–1500)	0.57
Intraoperative blood loss (mL)	50 (50–100)	50 (50–100)	0.88
Intraoperative urine output (mL)	350 (200–700)	300 (200–600)	0.52
Extubation time (min)	24 ± 9	26 ± 10	0.26
Recovery time (min)	66 ± 14	71 ± 21	0.20

*Note:* Data are presented as mean ± standard deviation (SD), median (interquartile range, IQR), or number (%).

Abbreviations: ASA, American Society of Anesthesiologists; HADS, Hospital Anxiety and Depression Scale; PSQI, Pittsburgh Sleep Quality Index; tACS, transcranial alternating current stimulation.

Pain scores across the five time points in both groups are summarized in Table 2. GEEs analysis revealed that the group × time interaction effect was not statistically significant for either pain type (resting pain: Wald *χ* ^2^ = 1.89, d*f* = 4, *p* = 0.756; movement‐evoked pain: Wald *χ* ^2^ = 4.05, d*f* = 4, *p* = 0.400), indicating that the tACS group and sham group exhibited consistent overall trends in pain score changes over time. The tACS group demonstrated significantly lower overall postoperative rest pain scores compared to the sham group (*β* = −0.49, 95% confidence interval [CI], −0.78 to −0.20, *p* = 0.001). Similarly, the tACS group showed significantly lower overall movement pain scores than the sham group (*β* = −0.45, 95% CI, −0.84 to −0.06, *p* = 0.025) (Table 2).

**TABLE 2:** Comparison of postoperative pain scores between tACS and sham groups.

Pain type	Time point	tACS group	Sham group	GEE main effect (tACS vs. sham)
		*N* = 36	*N* = 35	
Pain score at rest, NRS (0–10)	2 h	2 (1–2)	2 (1–2)	*β* = −0.49, 95% CI, −0.78 to −0.20; *p* = 0.001
	6 h	2 (1–3)	2 (1–3)	
	12 h	1.5 (1–3)	2 (2–3)	
	24 h	1 (1–2)	2 (1–3)	
	48 h	1 (1–2)	2 (1–2)	

Pain score at movement, NRS (0–10)	2 h	3 (2–4)	3 (3–4)	*β* = −0.45, 95% CI, −0.84 to −0.06; *p* = 0.025
	6 h	3 (3–5)	3 (3–4)	
	12 h	3 (2–4)	4 (3–4)	
	24 h	3 (2–4)	4 (3–5)	
	48 h	3 (2–3)	3 (3–4)	

*Note:* Data are presented as mean ± standard deviation (SD), median (interquartile range, IQR), or number (%).

Abbreviations: CI, confidence interval; NRS, numeric rating scale; tACS, transcranial alternating current stimulation.

**FIGURE 2:**
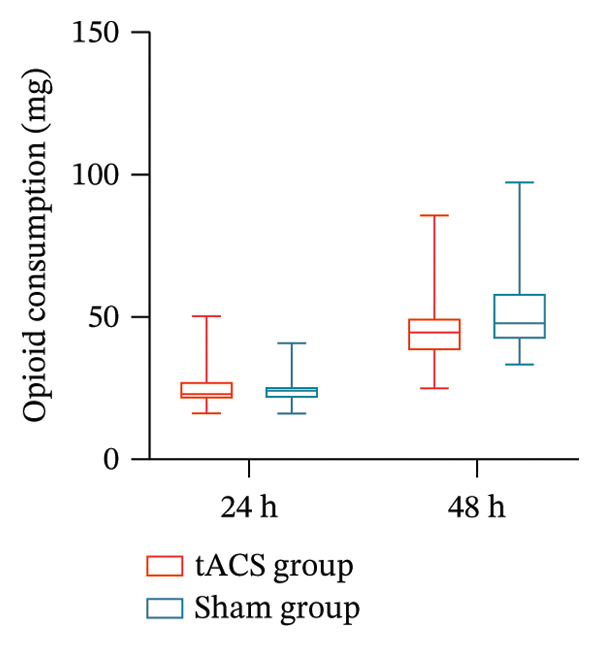
Between‐group comparisons of the cumulative opioid consumption during the 24 h and 48 h postoperatively. *Note:* The solid line represents the median, with the upper and lower quartiles at both ends of the column and the lowest and largest edges at the lower and upper edges.

No statistically significant difference was observed in cumulative opioid consumption between the two groups within 24 h postoperatively (median difference [MD] = 1.0 mg; 95% CI, −2.3 to 2.5; *p* = 0.76) (Table 3, Figure 2). In addition, the cumulative opioid dosage also did not differ significantly between groups during the 48 h postoperative period (MD = 4.0 mg; 95% CI, −0.6 to 9.1; *p* = 0.10) (Table 3, Figure 2). Additionally, the AUC for NRS pain scores over 24 and 48 h was further calculated, and it was found that AUC_2–24 h_ and AUC_2–48 h_ of resting pain and AUC_2–48 h_ of movement pain scores were lower compared to the sham group, with differences that were statistically significant (Table 3).

**TABLE 3:** Comparison of cumulative opioid consumption and AUC of pain between tACS and sham groups.

	tACS group	Sham group	Median difference, 95% CI	*p*
	*N* = 36	*N* = 35		
24‐h cumulative opioid consumption (mg)	23.0 (20.1–27.0)	24.0 (20.8–26.1)	1.0 (−2.3–2.5)	0.76
48‐h cumulative opioid consumption (mg)	44.6 (37.6–50.2)	47.8 (41.6–58.9)	3.3 (−0.6–9.1)	0.10
AUC_2–24 h_ of rest pain	38.5 ± 22.2	49.9 ± 18.4	11.4 (3.5–19.4)	0.005
AUC_2–48 h_ of rest pain	74.1 ± 26.6	99.6 ± 34.8	25.5 (10.8–40.1)	0.001
AUC_2–24 h_ of movement pain	71.7 ± 23.0	80.4 ± 22.2	8.7 (−2.0–19.4)	0.11
AUC_2–48 h_ of movement pain	139.4 ± 39.8	164.7 ± 43.7	25.4 (5.6–45.1)	0.013

*Note:* The values are presented as the median (interquartile range [range]) or number (%).

Abbreviations: AUC, area under the curve; CI, confidence interval; NRS, numeric rating scale; tACS, transcranial alternating current stimulation.

**FIGURE 3:**
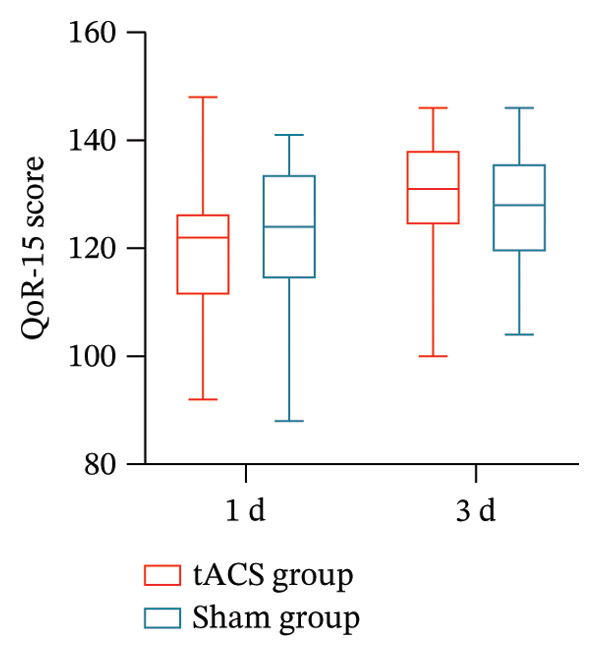
Between‐group comparisons of the Quality of Recovery‐15 (QoR‐15) score on postoperative Day 1 and Days 3. *Note:* The solid line represents the median, with the upper and lower quartiles at both ends of the column and the lowest and largest edges at the lower and upper edges.

The QoR‐15 scores were comparable between the two groups on postoperative Day 1 [122 (111–126) vs. 124 (114–134); MD = 3; 95% CI, −4–9; *p* = 0.46] and postoperative Day 3 [131 (124–138) vs. 128 (119–136); MD = −2; 95% CI, −8–3; *p* = 0.38] as shown in Figure 3.

The occurrence of analgesic‐related side effects postoperatively was not significantly different between the two groups (Table 4). Two patients in the tACS group reported scalp tingling, and none of the patients experienced moderate to severe electrical stimulation‐related complications.

**TABLE 4:** Analgesic‐related side effects.

	tACS group	Sham group	Risk ratio, 95% CI	*p*
	*N* = 36	*N* = 35		
Postoperative nausea and vomiting, *n* (%)	5 (13.9)	4 (11.4)	1.3 (0.3–5.1)	1.00
Dizzy, *n* (%)	8 (22.2)	8 (22.8)	1.0 (0.3–2.9)	0.95
Itching, *n* (%)	3 (8.3)	2 (5.7)	1.5 (0.2–9.6)	1.00
Drowsiness, *n* (%)	6 (16.7)	7 (20.0)	0.8 (0.2–2.7)	0.72

## 4. Discussion

The current study showed that among patients undergoing VATS, a single 20‐min session of tACS over primary somatosensory cortex (S1) led to a statistically significant yet clinically mild reduction of rest and movement pain scores. However, the tACS intervention did not reduce the postoperative opioid consumption at 24 and 48 h, improve the quality of postoperative recovery, or decrease the incidence of adverse reactions.

In this study, statistically significant differences between groups were observed in postoperative pain outcomes. To comprehensively quantify the overall pain burden during the early postoperative period, we further calculated the AUC of pain scores postoperatively. Specifically, the tACS group exhibited significantly lower AUC values for resting pain (both AUC_2–24 h_ and AUC_2–48 h_) and movement pain (AUC_2–48 h_) compared with the sham stimulation group. These findings indicate that α‐tACS could alleviate patients’ subjective pain experience and reduce postoperative pain scores. The result aligns with prior evidence from healthy volunteers [17] and patients with chronic low back pain [21]. The consistency of results underscores the translational potential of tACS as a nonpharmacological adjunct for acute pain management. Notably, statistical significance does not equate to clinical significance. Our main effect of group analysis showed that the reduction in postoperative pain scores was less than 1 point, falling short of the threshold for the minimum clinically important difference (MCID) in pain scores. This modest effect size highlights that single‐session tACS, as currently applied, has limited clinical utility as a standalone analgesic intervention. It is important to acknowledge that not all prior studies have consistently supported the analgesic effect of tACS. For instance, another research report stated that α‐tACS on the bilateral sensorimotor cortex had no effect on experimental thermal pain [24], and a systematic review noted contradictory results regarding tACS for chronic pain management [11]. This modest effect size, as well as the heterogeneity of tACS efficacy reported in the literature, may be attributed to multiple factors, including tACS parameters (e.g., stimulation frequency, intensity, and target site), pain model selection (e.g., differences between experimental and chronic pain), assessment methods, and individual pain sensitivity. Our study was designed to reflect real‐world clinical practice, where multimodal analgesia is the standard of care; thus, we aimed to evaluate the synergistic value of tACS as an adjunct rather than a replacement for pharmacological interventions. Given the clinical utility and potential effectiveness of a single brain stimulation technique in pain relief [25, 26], we initially chose to implement only a single stimulus as an intervention. However, research shows that multisession tACS can induce neuroplastic changes and further prolong and amplify therapeutic effects [27, 28]. Additionally, studies indicate that the dorsolateral prefrontal cortex (DLPFC) via descending pain modulation systems and the primary motor cortex (M1) via sensorimotor integration mechanisms may also be involved in pain regulatory networks [29], and multitarget combined stimulation of these brain regions may enhance the analgesic potential for postoperative pain. Overall, the role of tACS in postoperative acute pain management remains incompletely established, and future studies should explore the application value of repeated multisession tACS stimulation, different stimulation targets, and combined stimulation.

Notably, no significant differences in postoperative opioid consumption were observed between the two groups at 24 and 48 h postoperatively. The discrepancy between reduced pain scores and unchanged opioid consumption can be explained by three main factors. First, the single‐session tACS protocol may have been insufficient to achieve a robust analgesic effect capable of reducing opioid requirements. Second, the mild magnitude of pain reduction was insufficient to trigger opioid dosage adjustments in the context of multimodal analgesia. Third, postoperative opioid consumption is regulated by multiple factors, including patients’ psychological characteristics, interindividual variability in analgesic drug sensitivity, and institutional standardized perioperative analgesic regimens [30, 31], which may mask the opioid‐sparing potential of α‐tACS. In this study, preoperative paravertebral nerve block, as a component of multimodal analgesia, may have contributed to a distinct floor effect. Satisfactory pain control could keep postoperative pain scores and analgesic demands at low levels, leaving limited room for further opioid reduction and hindering the detection of tACS‐related opioid‐sparing effects.

Our study also found no significant difference in postoperative recovery quality assessed using the QoR‐15 scale between groups. The QoR‐15 scale is a patient‐centered self‐rating scale. Yoon et al. showed postoperative pain intensity has a significant correlation with the quality of recovery as assessed by the QoR‐15 [32]. Although there was a statistically significant difference in pain at rest in this study, this is not unexpected given that postoperative pain intensity was in the mild to moderate range. Regarding safety, low‐intensity transcranial electrical stimulation is currently considered safe [33]. Mild adverse effects mainly include tingling and burning sensation during tDCS with a peak intensity of 1–2 mA and during tACS with a peak intensity above 2 mA, as well as headache and fatigue after stimulation. Moderate adverse events such as skin burns are also rare, and no serious adverse effects have been reported. In our study, only two patients reported scalp tingling with no other adverse effects.

Our study has several strengths. To the best of our knowledge, it is the first randomized controlled trial exploring tACS for VATS postoperative acute pain. Second, we enrolled patients from the same group of surgeons to reduce bias in surgical procedures and postoperative management, and we used sham stimulation for the control group to minimize the placebo effect. We also excluded patients with known factors influencing postoperative pain, such as preoperative chronic pain and long‐term opioid use. Preoperative anxiety and depression were assessed, and no significant differences between groups were found. Nevertheless, our study has some limitations. First, the pain reduction was modest and did not reach MCID, limiting the immediate clinical utility of single‐session tACS. Second, all participants underwent preoperative paravertebral nerve block, which may produce a floor effect and weaken the measurable analgesic benefit of tACS. In addition, only single‐session tACS intervention and single‐target stimulation (S1) were adopted. These factors contributed to the relatively mild analgesic effects observed in our trial. Third, analgesic efficacy was assessed using subjective measures only, and the lack of objective neurophysiological or neuroimaging data limits the robustness of our findings and prevents verification of potential mechanisms. Fourth, although sham stimulation was employed, no formal assessment of blinding success was conducted. Overall, these limitations indicate that the results should be interpreted as preliminary, and further research is warranted.

## 5. Conclusion

This study demonstrates that incorporating tACS into a multimodal analgesia regimen yields a statistically significant yet modest reduction in postoperative pain among VATS patients, without reducing overall postoperative opioid consumption. As this represents a preliminary study on the use of tACS for perioperative pain management, further research is required to clarify its clinical impact and value before this approach can be widely adopted in clinical practice.

**Nomenclature**
AUC: Area under the curve
DLPFC: Dorsolateral prefrontal cortex
M1: Motor cortex
MD: Median difference
NRS: Numerical rating scale
QoR‐15: Quality of recovery‐15
S1: Primary somatosensory cortex
tACS: Transcranial alternating current stimulation
tDCS: Transcranial direct current stimulation
VATS: Video‐assisted thoracoscopic surgery

## Author Contributions

Jin‐Jin Yang: conceptualization; methodology; writing–original draft. Sai Chen: conceptualization; formal analysis; writing–review and editing. Zhi‐Hao Li: investigation; writing–review and editing. Ni Du: investigation; writing–review and editing. Lei Lei: conceptualization, methodology; writing–review and editing; supervision. Jian‐Jun Yang: conceptualization, methodology; data curation; writing–review and editing; supervision; funding acquisition.

## Funding

This work was supported by the National Natural Science Foundation of China (82171189).

## Ethics Statement

The trail was approved by the Ethics Review Committee (2023‐KY‐1142‐003) of the First Affiliated Hospital of Zhengzhou University based on the Declaration of Helsinki. Written consent was obtained from each patient before surgery.

## Conflicts of Interest

The authors declare no conflicts of interest.

## Data Availability

The data that support the findings of this study are available from the corresponding author upon reasonable request.
